# Sex-Differences in Health-Related Characteristics of Senior Center Users: The VERISAÚDE Study

**DOI:** 10.3389/fpsyg.2020.00964

**Published:** 2020-05-15

**Authors:** Laura Lorenzo-López, Rocío López-López, Ana Maseda, Ana Buján, José Luis Rodríguez-Villamil, José Carlos Millán-Calenti

**Affiliations:** Gerontology Research Group, Instituto de Investigación Biomédica de A Coruña (INIBIC), Complexo Hospitalario Universitario de A Coruña (CHUAC), SERGAS, Universidade da Coruña, A Coruña, Spain

**Keywords:** older adults, health, sex-related differences, women characteristics, multidimensional assessment

## Abstract

**Background:**

We explored sex-related differences in sociodemographic, medical, psychological, and functional conditions in older adults attending to senior citizens’ centers.

**Materials and Methods:**

An exploratory study was conducted as part of the VERISAÚDE project, a cross-sectional population-based study of individuals aged ≥65 years enrolled in senior community centers located in Galicia, Northwest of Spain (*n* = 749). A comprehensive gerontological evaluation was used to assess the social, medical, psychological, and functional characteristics of the sample.

**Results:**

Women presented a higher prevalence of frailty (*p* = 0.017), a higher risk of malnutrition (*p* = 0.029), more medication consumption (*p* = 0.002), and polypharmacy (*p* = 0.008), higher depressive scores (*p* = 0.007), and lower cognitive scores (*p* = 0.045) than men, who showed a higher prevalence of hearing impairment (*p* = 0.034), toxic habits (all ps = 0.0001), and comorbidity (*p* = 0.002), and better quality of life (*p* = 0.030), and social resources (*p* = 0.002). Participants considered that attending and being involved in senior centers has a positive influence on their health and promotes successful aging.

**Discussion:**

Important differences were found between women and men in health variables, suggesting that sex exerts a powerful influence on health status in older age. These differences should be identified and taking into account when designing interventions to promote active aging and to improve the quality of life of older adults. Taking a sex perspective during the evaluation process could lead to a higher number of older people being effectively treated in clinical practice.

## Introduction

The health status of older adults is highly variable, with some individuals experiencing few age-related limitations and others facing with multiple comorbidities, social isolation, and reduced socioeconomic resources. Additionally, men and women might face distinct challenges and stressors in late life, and they might experience different and specific physical and mental health problems. Indeed, sex has been recognized as a variable that influences behavioral and lifestyle choices and, consequently, may play an important role in the development and progression of age-related chronic diseases. Importantly, the known universal women advantage in life expectancy does not necessarily mean that they present better health status than men. In fact, higher prevalence of functional limitations, physical disability, and poor self-rated health has been reported among women compared with age-matched men in previous studies (von Strauss et al., 2003; Murtagh and Hubert, 2004; Christensen et al., 2009). In general, sex differences in health status in late life have been explained attending to psychosocial factors, such as differential vulnerability, and/or exposure to social and biological factors among women and men. However, sex differences in general health status have not been previously explored in an older community-dwelling Galician population, and there is still much to be done in order to further understand why men and women age in different ways, and to identify the principal factors that may explain the sex differences in late-life health.

The singular demographic characteristics of Galicia, located in the south of Europe (northwestern region of Spain), with a high index of older citizens, high feminization of aging, and dispersed population, makes this region especially optimal to explore sex differences in health-related outcomes. In this context, Galicia has been recognized by the European Commission (EC) as a Reference Site in the European Innovation Partnership on Active and Healthy Aging and its impact in Spain (García Lizana et al., 2018).

While there are published studies describing differences between older men and women in specific health outcomes and indicators (Guallar-Castillón et al., 2005; Alvarado et al., 2007; Etherington, 2017), studies that have specifically examined sex differences in general health status by a comprehensive approach including the multidimensional assessment of several domains are more limited (Ferrer et al., 2011). Previous studies have documented sex-based inequalities in both physical and mental health (Murtagh and Hubert, 2004; Kendler et al., 2005; Alvarado et al., 2007; Munro et al., 2012), in self-reported health status (Etherington, 2017), and in quality of life (Guallar-Castillón et al., 2005; Orfila et al., 2006), which has been considered as an indicator of overall health and medical needs, and as a consistent independent predictor of mortality. In general, the results of these studies have highlighted the complexity of sex differences in late-life health. Based on this previous research and on the potential differential life course between men and women, we hypothesized that older men and women would differ in their general health status.

With the worldwide growth of the aging population, increasing our knowledge about the health status, survival, and specific care needs of older men and women is relevant for the future of the public health care system, and it will help to define individualized treatment and preventive interventions. Our objective was to explore and describe the general health status and sex-related differences in sociodemographic, medical, psychological, and functional conditions in a sample of older adults regularly and actively attending to senior centers. To this end, we employed a comprehensive gerontological evaluation, including the multidimensional assessment of various domains (physical/medical, functional, psychological, and social; Rubenstein, 2004; Elsawy and Higgins, 2011), by a multidisciplinary team of trained health professionals. Since many older adults suffer from multiple, complex, and interdependent health-related conditions (great medical complexity, major cognitive, affective, and functional problems), comprehensive assessment methodologies are particularly suited to explore their health situation and potential sex differences. We define health as a multidimensional concept encompassing all of these domains, as well as the self-rated or subjective health status. It has been demonstrated that the systematic evaluation of these domains may help to the early identification and analysis of potential treatable health problems or older care needs, leading to better health outcomes and specific interventions (Rubenstein, 2004). Developing a comprehensive assessment would help to guide decision-making and to incorporate potential sex differences in health outcomes into treatment decisions, in order to maximize overall health with aging. This is a crucial point since despite the potential differences between the two sexes; there is little sex-specific health care strategies and policies in geriatric clinical medicine.

## Materials and Methods

### Selection and Description of Participants

The study was conducted as part of the VERISAÚDE project, a cross-sectional population-based study of community-dwelling individuals aged ≥ 65 years, residing in Galicia. Participants were recruited from 43 senior community centers. The statistical parameters adopted for the calculation of the initial sample size were: maximum statistical error of 4.0% for Type I and 20.0% for Type II, with a power of 95.0%. Considering the absolute number of Galician older adults aged ≥65 years according to the municipal register of the 2011 National Health Survey (632,381 individuals), a sample of 749 older individuals was defined, with the age and sex distribution similar to that of the older population of Galicia (see Lorenzo-López et al., 2017).

The study protocol was approved by the Ethics Committee of the University of A Coruña and was in conformity with the principles embodied in the Declaration of Helsinki. Before data collection, all participants were informed about the project and signed the corresponding informed consent form. The manuscript was written according to the STrengthening the Reporting of OBservational Studies in Epidemiology (STROBE) statement (Vandenbroucke et al., 2014).

### Procedure

From October 2013 to March 2014, participants were individually assessed in the senior centers by a multidisciplinary team of professionals with experience in gerontological assessment (clinical psychologists, nurses, occupational therapists, and social workers). The assessment process included information on sociodemographic data, social resources, sensory impairments, toxic habits, medications history, and polypharmacy, comorbidity, nutritional status, self-rated health, general cognitive function, depressive symptoms, quality of life, instrumental activities of daily living (IADL), and frailty status. The specific inclusion criteria were: (1) being ≥65 years old, (2) being actively enrolled in a senior center, and (3) willingness to sign the informed consent form. The exclusion criterion was the inability to fully comprehend and/or perform the assessment instruments.

### Variables and Instruments

#### Sociodemographic and Social Resources Assessment

Date of birth, age, sex, and education level were self-reported. Educational level was categorized into three groups according to completed years of formal education: ≤8 years, 9–17 years, and ≥18 years.

Social resources were measured by the Spanish version (Grau Fibla et al., 1996) of the Older Americans Resources and Services (OARS) scale (Fillenbaum, 1988), consisting of 9 items. To rate the adequacy of social support three psychometric factors were extracted reflecting: (1) the availability and amount of contact with others (interaction dimension of social support: item 3 “Number of people known well enough to visit,” item 4 “Times talking with someone on telephone per week,” and item 5 “Times visiting with someone per week”); (2) the adequacy of contacts (affective dimension of social support: item 6 “Have someone you trust,” item 7 “Frequency of feelings of loneliness,” and item 8 “Satisfaction with contacts with loved ones,” and (3) the availability of close support (dependability dimension of social support: item 9 “Have someone who would help you”). The information from these three factors were combined into a 6-point summary rating of social resources using the SPSS (IBM SPSS Statistics 23.0; IBM Corp., Armonk, NY, United States) program statements provided by Fillenbaum (1988): (a) excellent, (b) good, (c) mild impairment, (d) moderate impairment, (e) severe impairment, and (f) total impairment.

A questionnaire was administered to the participants to assess the role that senior centers play in their lives. It asked for perceived benefits of attending (promoting active aging, improving self-esteem, improving social relationships, and improving physical and psychosocial health).

#### Medical Assessment

The medical dimension covered in the evaluation process included the assessment of sensory impairments, toxic habits, medication consumption, comorbidity, and nutritional status of the participants. These variables might have considerable impact on function and affect well-being of older adults.

##### Visual and hearing sensory impairments

To identify hearing impairment, the whispered-voice test was used (Swan and Browning, 1985), with the examiner standing 6 m behind the seated participant. This simple voice test reliably identifies individuals with a hearing disability (Swan and Browning, 1985). The hearing was considered normal if the participant repeats back correctly at least 3 out of a combination of 6 letters and numbers. To assess the visual acuity, a Snellen chart was placed at a distance of 2.8 m from participant’s eyes. This visual test is simple to perform and is sensitive to visual impairment. A decreased visual acuity was defined as the best corrected vision worse than 20/50 (Snellen, 1868).

##### Toxic habits (tobacco and alcohol consumption)

Self-reported tobacco smoking and alcohol consumption patterns were recorded. We considered the 30 days prevalence of cigarette smoking (Lantz, 2003) to categorize the smoking status (smoker or non-smoker). We calculated the number of Standard Drink Units (SDU) with the formula: size of drink in milliliters (Vol) x percent by volume of alcohol (%) x density of ethanol at room temperature (0.789 g/ml)/by the grams in the standard drink (10 grams in Spain), and defined “alcohol abuse” as a daily consumption > 30 grams of pure alcohol (3 SDU) per day (Foster and Marriott, 2006).

##### Medication consumption and polypharmacy

Participants were required to present their medication history, and polypharmacy was defined as the simultaneous use of ≥5 different prescribed medications (Gnjidic et al., 2012). This cutoff point has been shown to be valid to estimate the medication-related adverse effects in community-dwelling older adults (Gnjidic et al., 2012).

##### Comorbidity

Comorbidity was measured with the Charlson Comorbidity Index (CCI; Charlson et al., 1987), which provides a readily applicable and valid method of estimating risk of death from comorbid diseases. Patients were classified into 3 groups: 0–1 = no comorbidity; 2 = low comorbidity; and ≥3 = high comorbidity.

##### Nutritional status

We used the Spanish version of the Mini-Nutritional Assessment-Short Form (MNA-SF, Kaiser et al., 2009; Nestlé Nutrition Institute, 2009) for nutritional screening. This short form has been shown to be valid and to have good reliability (Kaiser et al., 2009). A score of 12–14 points corresponds to normal nutritional status, 8–11 points indicate risk of malnutrition, and 0–7 points indicate malnutrition.

#### Psychological Assessment

The psychological dimension of the evaluation process included the assessment of self-rated health, cognitive and affective status, and quality of life. The identification of psychological problems is crucial to obtain a complete picture of the health status of the participants.

##### Self-rated health

A single question was asked to evaluate self-rated health: In general, would you say your health is excellent, good, fair, or poor? (Kanagae et al., 2006).

##### Cognitive and affective assessment

The general cognitive status was evaluated with the Spanish version (Blesa et al., 2001) of the Mini-Mental State Examination (MMSE, Folstein et al., 1975), which complies with the psychometric requirements of reliability and reproducibility (Blesa et al., 2001). Scores range from 0 to 30, and were adjusted for level of education and age. Scores >25 points suggest cognitive impairment (Blesa et al., 2001).

The presence of depressive symptoms was evaluated with the Spanish-validated version (Martínez de la Iglesia et al., 2002) of the short-form of the Geriatric Depression Scale (GDS-SF; Sheikh and Yesavage, 1986), with a cut-off of ≥5 points (sensitivity 81.1%, specificity 76.7%; Martínez de la Iglesia et al., 2002), and indicating the probable existence of clinical depression. The parameters of validity and reliability for the Spanish version of the scale were similar to those of the original questionnaire (Martínez de la Iglesia et al., 2002).

##### Quality of life

We used the Spanish version (Lucas-Carrasco, 1998) of the World Health Organization’s Quality of Life measure-brief version (WHOQOL-BREF) instrument (Skevington et al., 2004) to assess the quality of life. It has been shown to display good discriminant validity, content validity, and test-retest reliability. It comprises 26 items, which measure 4 major domains: Physical health, psychological health, social relationships, and environment. Each domain is scored on a five-point Likert scale (higher scores denoting the higher self-rated quality of life). The mean score of items within each domain is used to calculate the domain score. It also produces two separated general scores relating to the individual’s overall perception of quality of life and general health.

#### Functional Assessment

We used the Lawton’s (IADL; Lawton and Brody, 1969) scale to evaluate potential functional dependence to perform instrumental activities of daily living. This is a valid and reliable scale to assess independent living skills in non-institutionalized older adults. Participants were asked if they had difficulty in performing 8 instrumental activities with no help. The score range is 0 to 8. Individuals who were unable to perform any one of the activities were considered functionally incapacitated.

Frailty phenotype was assessed using Fried et al. (2001) criteria: (a) unintentional weight loss of ≥4.5 kg in last year, (b) self-reported exhaustion, (c) weakness, defined by handgrip strength in the dominant hand measured with a dynamometer in kilograms, adjusted for sex, and body mass index, (d) slowness, assessed by the walking time (in seconds) over a distance of 4.57 m, adjusting for sex, and height, and (e) low physical activity, measured by the weighted score of kilocalories expended per week, based on each participant’s report, and adjusting for sex. Frailty phenotype has been shown to be a valid method for frailty screening (Fried et al., 2001).

### Statistical Analysis

Sample characteristics are presented as mean (SD) for continuous variables and as a percentage (n,%) for categorical variables. The normality of the data was tested using the Kolmogorov–Smirnov test, which rejected the assumption of normality, but the sample size was sufficiently large to apply parametric instead of non-parametric tests. Student *t*-test was used to assess sex differences in means, and Chi-square test was used to assess sex differences in proportions. The column proportions were compared using a *Z* test for multiple response variables; with the *p* values adjusted using the Bonferroni method. Effect sizes (ES) were estimated in terms of Cohen’s (1988) *d* to identify differences in group mean values and to determine their clinical significance. “Small ES” (*d* = 0.2), “medium ES” (*d* = 0.5), and “large ES” (*d* = 0.8) benchmarks were used to interpret the ES magnitude. For difference among proportions Cohen’s (1988) *h* was calculated, considering “small ES” (*h* = 0.2), “medium ES” (*h* = 0.5), and “large ES” (*h* = 0.8) benchmarks. All statistical tests were performed using IBM SPSS Statistics 23.0 (IBM Corp., Armonk, NY, United States). The level of significance was defined as *p* < 0.05.

## Results

### Sex-Related Differences in Socio-Demographic and Social Resources Variables

The mean age of the participants was 75.8 ± 7.2 years (range, 65–97 years) and most were women (454, 60.6%).

Women were slightly older than men (women 76.2 ± 7.3 years, men 75.0 ± 6.9 years, *t*(747) = 2.189, *p* = 0.029, and *d* = 0.169), but there were no significant sex differences in education level [χ2(2, *n* = 749) = 1.40, *p* = 0.497], and with most of the participants having completed ≤8 years of education (61.7% women and 58.0% men).

**FIGURE 1:**
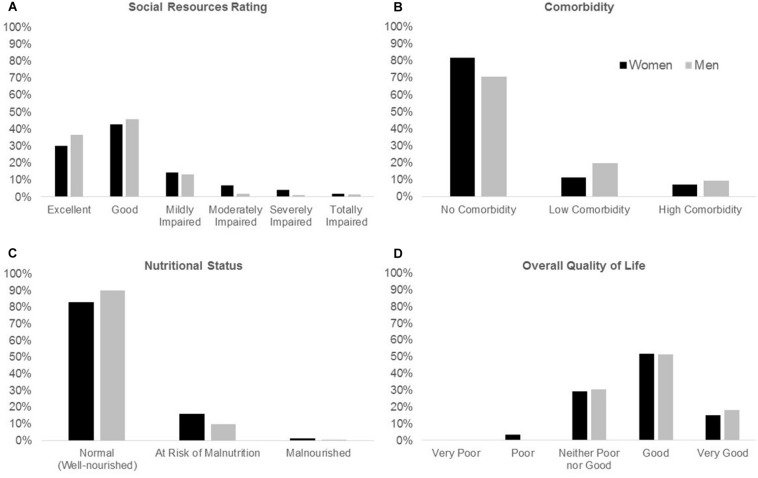
Sex differences in social resources rating **(A)**, comorbidity **(B)**, nutritional status **(C)** and overall quality of life **(D)**.

Regarding social resources (see Figure 1A), 43.9% of the respondents had good social resources, and 32.7% had excellent social resources. Only 1.6% of the participants reported totally impaired social resources. Statistical analyses revealed significant sex differences [χ2(5, *n* = 731) = 18.53, *p* = 0.002], with women having poorer social resources. In fact, according to the 6-point classification, women showed moderate and severe impairment in their social resources more frequently than men (moderate impairment: 6.8% women vs 1.7% men, *h* = 0.252; severe impairment: 4.1% women vs 1.0% men, *h* = 0.203).

Participants rated the importance of their participation in the centers’ activities as very high: 8.78 out of 10 (women: 8.98; men: 8.49). Specifically, 98% of them considered that regularly attending to the center promotes active aging (women: 97.9%; men: 98.2%). 66.3% believed that their social relationships improved (women: 69.2%; men: 61.9%), and 52.3% considered that their physical and psychosocial health improved since regularly participating in the activities (women: 58.3%; men: 42.8%).

### Sex-Related Differences in Medical Variables

In the whole sample, 8.6% of the participants presented visual impairment and 27.9% showed hearing loss. No significant sex differences in the prevalence of visual impairments [χ2(1, *n* = 736) = 1.81, *p* = 0.179] were observed, but hearing impairment was more frequent in men [women 25.1%, men 32.2%, χ2(1, *n* = 749) = 4.47, *p* = 0.034, and *h* = −0.156].

Toxic habits were also more frequent in men [tobacco consumption: χ2(2, *n* = 748) = 228.53, *p* < 0.0001, *h* = −0.536; grams of pure alcohol per day: *t*(401) = −4.372, *p* < 0.0001, *d* = −0.431; and alcohol abuse: χ2(1, *n* = 749) = 42.33, *p* < 0.0001, *h* = −0.476].

The mean number of consumed medications was 4.8 ± 3.3, and it was significantly higher in women than in men [women 5.1 ± 3.3, men 4.3 ± 3.2, *t*(747) = 3.075, *p* = 0.002, *d* = 0.246). As a result, polypharmacy was also higher in women (women 52.0%, men 42.0%, χ2(1, *n* = 749) = 7.090, *p* = 0.008, and *h* = 0.201].

Of the whole sample, 77.4% of participants showed no comorbidity, 14.6% showed low comorbidity, and 8.0% showed high comorbidity, with significant differences as a function of sex [χ2(2, *n* = 749) = 12.79, *p* = 0.002]. In women, the proportions of no comorbidity, low comorbidity, and high comorbidity were 81.7, 11.2, and 7.0%, respectively. Corresponding percentages in men were 70.8, 19.7, and 9.5% (see Figure 1B). Column comparisons revealed that the proportion of no comorbidity was higher in women (*h* = 0.261) and that the proportion of low comorbidity was higher in men (*h* = −0.251).

Finally, 85.7% of the participants showed a normal nutritional status (see Figure 1C), 13.5% were at risk of malnutrition, and only 0.8% were malnourished. Importantly, malnutrition risk was significantly higher in women than in men [χ2(2, *n* = 749) = 7.08, *p* = 0.029].

### Sex-Related Differences in Psychological Variables

Statistical sex-related differences were not identified on self-rated health [χ2(3, *n* = 748) = 6.16, *p* = 0.103]. Of the whole sample, 22.1% of the participants rated their health as excellent, 56.1% as good, 19.0% as fair, and 2.8% as poor.

The prevalence of cognitive impairment was 6.5%. Although the mean of MMSE scores was slightly greater in men [women 28.2 ± 2.5, men 28.5 ± 1.9, *t*(747) = −2.004, *p* = 0.045, *d* = −0.135], there were no significant sex differences in the prevalence of cognitive impairment [χ2(1, *n* = 749) = 3.63, *p* = 0.057].

The prevalence of depressive symptoms was 8.1%. Women scored significantly higher on depressive symptoms than men [women 1.7 ± 2.2, men 1.3 ± 1.8, *t*(747) = 2.689, *p* = 0.007, and *d* = 0.199], but there were no significant differences in the prevalence of depressive symptomatology [χ2(1, *n* = 749) = 1.89, *p* = 0.169].

Regarding the quality of life, men rated their overall quality of life significantly higher than women [χ2(1, *n* = 749) = 10.73, *p* = 0.030, see Figure 1D] and they tended to be more satisfied with their health than women, although this difference was not significant [χ2(1, *n* = 749) = 4.41, *p* = 0.353]. Physical health, psychological, and environment mean domain scores of the WHOQOL-BREF were higher in men than in women (*p* < 0.0001, *d* = −0.389; *p* = 0.001, *d* = −0.256; and *p* = 0.048, *d* = −0.166, respectively), indicating better quality of life in men in these domains. However, social relationships scores were higher in women than in men (*p* = 0.038, *d* = 0.157). Over half (51.5%) of the total sample reported “good” levels of quality of life, and 56.6% were satisfied with their health.

### Sex-Related Differences in Functional Variables

The prevalence of dependence regarding instrumental activities of daily living was 12.4% in the total sample, with no significant sex differences [χ2(1, *n* = 749) = 3.60, *p* = 0.058]. The prevalence of frailty syndrome was 3.7%, with a higher prevalence among women [women 5.1%, men 1.7%χ, 2(1, *n* = 749) = 5.65, *p* = 0.017, and *h* = 0.167].

## Discussion

In the present paper, we used a comprehensive gerontological evaluation to describe the health status and to estimate sex-related differences of independently living older adults, actively participating in senior centers from Galicia. The demographic characteristics of this region from Europe makes it especially optimal to explore sex-related differences in health-related outcomes. Results revealed significant differences in various domains between sex groups, suggesting that sex exerts a powerful influence on health status in older age.

### Sex-Related Differences in Sociodemographic and Social Resources Variables

Although women were slightly older than men, it is not likely that this difference explains sex differences in the rest of the explored outcome variables.

Regarding social resources, the mean rating showed that participants’ social resources were good or excellent, and women had poorer perceived social resources than men. In this line, being women has been associated with social risk in a previous multicenter study exploring sex differences in health status in a non-institutionalized Spanish population aged 85 or over (Ferrer et al., 2011). This is a relevant point since women have been shown to be more sensitive than men to the depressogenic effects of low level of social support (Kendler et al., 2005).

Participants considered that attending and being involved in senior centers has a positive influence on their health and promotes successful aging.

### Sex-Related Differences in Medical Variables

No significant sex differences in the prevalence of visual impairment were observed. However, according to previous studies, hearing impairment was more frequent in men (Puts et al., 2005).

Toxic habits were also more frequent in men. Both tobacco and daily alcohol consumption were significantly higher among men, according to previous reports (Murtagh and Hubert, 2004). In the present study, daily alcohol intakes in grams of pure alcohol were on average 26.4 ± 18.6 grams for men and 19.5 ± 12.9 grams for women. Alcohol abuse is an important risk factor for many health problems (infectious diseases, cancer, diabetes, neuropsychiatric diseases, cardiovascular disease, liver and pancreas disease, and unintentional and intentional injury, Rehm, 2011) and, thus, is a major contributor to the global burden of disease (World Health Organization [WHO], 2014). These findings highlight the need to develop sex-specific effective prevention efforts to reduce alcohol abuse and its associated costs.

The mean number of consumed medications was higher in women than in men, as in previous studies (Murtagh and Hubert, 2004). Consequently, the prevalence of polypharmacy was 48.1%, with significant sex-related differences revealing higher prevalence in women (52.0% vs 42.0%). Polypharmacy has been previously associated with being women (Venturini et al., 2011), and it might have negative consequences for older adults, with drug-drug interactions, and adverse drug reactions.

Regarding comorbidity, significant differences were found as a function of sex. Specifically, the proportion of no comorbidity was higher in women and the proportion of low comorbidity was higher in men. In this line, being men was previously associated with higher comorbidity in a Spanish older population (Ferrer et al., 2011).

Regarding nutritional status, most of the participants were well nourished, and 13.5% were at risk of malnutrition. In previous studies, a relatively high prevalence of malnutrition risk has been reported in community-dwelling older adults (24.0–27.4%, Guigoz, 2006; Cereda, 2012), revealing malnutrition as an important health problem among older adults. It is important to note, that malnourished older people are more likely to require health and social services. Our prevalence rates of malnutrition risk were slightly lower than those reported in previous reviews, possible due to different mean age and criteria used to select participants. Importantly, malnutrition risk was significantly higher in women (Ferrer et al., 2011; Maseda et al., 2017).

### Sex-Related Differences in Psychological Variables

Significant sex differences were not observed in self-rated health, with the majority of participants rating their own general health as good. According to this finding, it has been reported that sex differences in self-rated health disappear by late adulthood (McCullough and Laurenceau, 2004). A recent study concluded that sex differences observed in self-rated health in some previous studies may actually be an artifact of the cohort (Etherington, 2017).

Regarding cognitive status, the prevalence of cognitive impairment was 6.5%, with no significant differences between sexes. However, our findings are based on a measure of general cognitive status, and we cannot rule out the possibility that sex differences exist in some cognitive domains but not in others. In fact, it has been reported that women perform better than men on tests of psychomotor speed and verbal learning and memory, whereas men perform better than women on visuospatial tests (Munro et al., 2012).

Although women in the general population suffer from depressive symptoms more frequently than men, few studies investigated sex differences in depressive symptomatology in the older people (Kockler and Heun, 2002; see Luppa et al., 2012 for a review; Zunzunegui et al., 1998). The prevalence of depressive symptoms is generally higher among older women than among men (Luppa et al., 2012), and depression in the older adults presents with a different distribution of symptoms in both sexes (Kockler and Heun, 2002). The higher prevalence of depressive symptomatology in women has been mainly explained attending to cumulative life course exposure to social and material disadvantages, and current socioeconomic, health condition, and functional disabilities (Alvarado et al., 2007). The reported prevalence rate of depressive symptoms among healthy older people living in the community was about 3.0% in previous studies (Lebowitz et al., 1997). The prevalence of depressive symptoms observed was higher; 8.1% overall. Importantly, although women scored slightly higher on the GDS-SF, there were no significant sex differences in the prevalence of depressive symptoms. In this regard, it has also been demonstrated that the difference in rates of depressive symptoms between women and men becomes progressively smaller with advancing age (Blazer and Williams, 1980).

Finally, regarding the quality of life, which provides a subjective overview of the health status, men rated their overall quality of life significantly higher than women, and they tended to be more satisfied with their own health. Sex differences in health-related quality of life have been previously explained attending to sociodemographic and potentially modifiable lifestyle factors (Guallar-Castillón et al., 2005) and to the prevalence of disability and chronic conditions (Orfila et al., 2006). The quality of life has also been associated with social network variables (Bergland et al., 2016) in community-dwelling older women. In this line, worse reported quality of life in older women in the present study may be partially explained by their poorer perceived social resources.

### Sex-Related Differences in Functional Variables

No significant sex-related differences in functional limitations on instrumental activities of daily living were observed in the present study. This result is in contrast with previous studies indicating that older women consistently report more functional limitations on IADL and have greater degrees of disability than age-matched men, mainly due to disability-related health conditions (Murtagh and Hubert, 2004).

The prevalence of frailty was 3.7%, which is relatively lower than the observed in previous cohort studies covering Spanish older adults (8.4% Abizanda et al., 2011; 16.9% Garcia-Garcia et al., 2011; and 9.6% Jürschik Giménez et al., 2011). This inconsistency may be due to differences in participant samples, and different procedures to define and assess frailty. The VERISAÚDE sample included community-dwelling older adults regularly and actively attending senior centers, while previous Spanish studies included institutionalized participants (Abizanda et al., 2011; Garcia-Garcia et al., 2011). These centers generally offer a variety of social and recreational programs and services. It has been shown that the regular participation in these activities enhances the overall health and well-being of older adults (Bugallo Carrera et al., 2014), which may contribute to avoid frailty syndrome (Gale et al., 2017). According to available literature, the prevalence of frailty was higher in women than in men (Collard et al., 2012; see Gordon et al., 2017 for a recent systematic review and meta-analysis; Puts et al., 2005).

The main limitation of this study is its descriptive, exploratory and cross-sectional nature, that it does not allow establishing specific causal relationships between sex and the studied variables. It is also important to highlight that in the present study only older adults actively participating in senior centers’ activities were evaluated, limiting the generalizability of the findings to all the community-dwelling older population from Galicia. Finally, since biological sex and sex may interact with each other to influence health, specific factors linked to sex (gene expression from the sex chromosomes, sex hormones, and metabolism of drugs by sex-specific cytochrome expression) should be studied in future works to develop appropriate strategies for health promotion.

To sum up, we detected important sex differences in health variables. Whether these dissimilarities are due to the different level of exposure to risk factors in women and men, or if they may result from a different sex-related reaction to the same factors should be further investigated in future longitudinal studies. Findings confirm that a comprehensive assessment is a valuable multidimensional diagnostic tool to detect, measure and manage the needs of community-dwelling older adults. Sex-related factors should be specifically addressed and integrated as interplaying factors in the context of the evaluation, and when developing effective and individualized health promoting interventions for older adults. It is also important considering the role of the changing environment and social/cultural circumstances in the physical and mental wellbeing of older men and women. The evaluation process should include further planning and follow-up of the individuals evaluated.

Efforts from the senior community centers’ coordinators should be directed to create public informative campaigns and to promote interventional programs taking into account specific differences and commonalities between older men and women. Major efforts should be made to highlight the importance of regular screening of physical and mental disorders to prevent the incidence of severe complications such as frailty syndrome and depressive symptoms in older women, and promoting the engagement of sex-specific health behaviors. Increasing awareness of sex-related differences in health and of positive lifestyle changes that can be adopted by older men and women (including regular physical and cognitive training, healthy nutrition, active living, and stress reduction) can lead to successful outcomes.

## Data Availability Statement

The raw data supporting the conclusions of this article will be made available by the authors, without undue reservation, to any qualified researcher.

## Ethics Statement

The studies involving human participants were reviewed and approved by the Ethics Committee of the University of A Coruña. Edificio Administrativo San Lázaro s/n. 15781 Santiago de Compostela. A Coruña. The patients/participants provided their written informed consent to participate in this study.

## Author Contributions

LL-L made substantial contributions to the study’s conception and design, actively participated in acquisition of data, analysis, and interpretation of data, and drafted the manuscript. RL-L actively participated in acquisition of data, analysis, and interpretation of data, and drafted the manuscript. AM and JM-C made substantial contributions to the study’s conception and design, actively participated in the interpretation of data, and revised the manuscript critically for important intellectual content. AB and JR-V revised the manuscript critically for important intellectual content.

## Conflict of Interest

The authors declare that the research was conducted in the absence of any commercial or financial relationships that could be construed as a potential conflict of interest.
